# PC1/3 KD Macrophages Exhibit Resistance to the Inhibitory Effect of IL-10 and a Higher TLR4 Activation Rate, Leading to an Anti-Tumoral Phenotype

**DOI:** 10.3390/cells8121490

**Published:** 2019-11-22

**Authors:** Franck Rodet, Alice Capuz, Bilgehan-Aybike Ozcan, Rémy Le Beillan, Antonella Raffo-Romero, Firas Kobeissy, Marie Duhamel, Michel Salzet

**Affiliations:** 1 Université de Lille, Inserm U1192–Laboratoire Protéomique, Réponse Inflammatoire et Spectrométrie de Masse (PRISM), F-59655 Villeneuve d’Ascq CEDEX, France; 2 Department of Psychiatry, McKnight Brain Institute, University of Florida, Gainesville, FL 32611, USA

**Keywords:** anti-tumoral immunotherapy, macrophages, proprotein convertase 1/3, TLR4 desensitization, IL-10 pro-tumoral effect

## Abstract

During tumorigenesis, macrophages are recruited by tumors and orientated towards a pro-tumoral phenotype. One of the main anti-tumoral immunotherapy consists of their re-polarization in an anti-tumoral phenotype. We have demonstrated that the inhibition of proprotein convertase 1/3 combined with TLR4 activation in macrophages is a promising strategy. These macrophages display pro-inflammatory and anti-tumoral phenotypes. A hallmark is a stronger activation of the pro-inflammatory NFKB pathway. We believe that this can be explained by a modification of TLR4 expression at the cell surface or MYD88 cleavage since it exhibits a potential cleavage site for proprotein convertases. We tested these hypotheses through immunofluorescence and Western blot experiments. A proteomics study was also performed to test the sensitivity of these macrophages to IL-10. We demonstrated that these macrophages treated with LPS showed a quicker re-expression of TLR4 at the cell surface. The level of MYD88 was also higher when TLR4 was internalized. Moreover, these macrophages were resistant to the pro-tumoral effect of IL-10 and still produced pro-inflammatory factors. This established that the sensitivity to anti-inflammatory molecules and the length of TLR4 desensitization were reduced in these macrophages. Therefore, during antitumoral immunotherapy, a repeated stimulation of TLR4 may reactivate PC1/3 inhibited macrophages even in an anti-inflammatory environment.

## 1. Introduction

During tumor development, cancer cells secrete chemokines such as CCL2, CCL5, CXCL12, and colony stimulating factor 1 (CSF1) to recruit immune cells towards the tumor site [1]. Tumors, therefore, exhibit a complex microenvironment consisting of a mix of tumor and immune cells. Among the immune cells present in the tumor microenvironment, tumor-associated macrophages (TAMs) play a critical role in tumor development. Indeed, tumor cells secrete anti-tumoral factors to create an immune-suppressive environment [2]. As a result, TAMs are orientated toward the anti-tumoral phenotype [3] and exert pro-tumoral functions. These functions include the promotion of angiogenesis [4], support of metastasis [5], suppression of anti-tumor immune response [6,7], secretion of growth factors supporting tumor growth [1], and resistance to therapy [1]. Undeniably, TAMs have been closely correlated with a poor prognosis in most types of cancer [8] and have, therefore, become a therapeutic target. To prevent the anti-tumor activities of TAMs, various therapeutic strategies have been developed such as blockage of macrophages recruitment, depletion of TAMs from the tumor, and inhibition of angiogenesis [1]. However, these approaches lead to various mechanisms of resistance [1]. To avoid this major drawback, the current strategy consists of re-polarizing the TAMs towards an anti-tumoral phenotype [9,10] in order to boost the immune response in cancer patients. 

In this context, we found that the endopeptidase proprotein convertase 1/3 (PC1/3) is a relevant target for anti-tumoral immunotherapy [11,12,13,14]. Indeed, PC1/3 knockdown macrophages (PC1/3 KD) present all the characteristics of activated pro-inflammatory macrophages [11,12,13,14,15,16]. These cells spontaneously secrete more pro-inflammatory cytokines and chemokines [11]. This effect was more prevalent after toll-like receptor 4 (TLR4) and toll-like receptor 9 (TLR9) stimulation [11,12,13]. It has been linked to a stronger activation of the pro-inflammatory pathway nuclear factor-kappa B (NFKB) and repression of the anti-inflammatory pathway signal transducer and activator of transcription 3 (STAT3) [11,12,13]. Noteworthy, this effect persisted even in the presence of an anti-inflammatory environment mimicked by interleukin-10 treatment [11,13]. The secretome from PC1/3 KD cells treated with lipopolysaccharides (LPS), a TLR4 ligand, exerted a strong chemotactic effect on human naïve CD4+T cells [11]. The nature of the cytokines and chemokines identified in these secretomes suggests that the naïve CD4+T cells could be orientated towards the TH1 pathway and, therefore, the cytotoxic response. Most importantly, after TLR4 stimulation, PC1/3 KD macrophages released killing factors that exerted a strong antitumor effect on breast (SKBR3) and ovarian (SKOV3) cancer cells [11]. We also found that extracellular vesicles isolated from PC1/3 KD cells challenged with the antitumor drug paclitaxel, a TLR4 sterile ligand, inhibited glioma growth [13]. Moreover, the supernatant collected from the co-culture between glioma cells and PC1/3KD macrophages also contained more anti-tumoral factors. In a tumor context, combining inhibition of PC1/3 and TLR4 activation in macrophages could, therefore, be a very promising strategy to reactivate the immune system and obtain cytotoxic effects against malignant cells. 

In the present paper, our aim is to understand at the molecular level how the absence of PC1/3 in macrophages enables a sustained activation of the TLR4 pathway and a resistance to the pro-tumoral effect of IL-10 treatment. We demonstrate that TLR4 trafficking from intracellular compartments towards the plasma membrane is altered in macrophages inhibited for PC1/3 and stimulated with LPS. As a result, TLR4 is more quickly re-expressed at the cell surface. We also found that the level of myeloid differentiation factor 88 (MYD88), the key TLR signaling adaptor, is higher in PC1/3 KD cells treated with LPS, whereas the receptor is internalized. These results suggest that the duration of TLR4 desensitization is reduced in PC1/3 KD macrophages. Therefore, these macrophages could be activated more frequently with a TLR4 ligand. Moreover, PC1/3 KD cells resist to the inhibitory effect of IL-10 and clearly release pro-inflammatory factors. These characteristics are clearly advantageous for anti-tumoral immunotherapy aiming to reactivate TAMs.

## 2. Materials and Methods

### 2.1. Reagents

The rat alveolar macrophage NR8383 cell line (CRL-2192) was obtained from ATCC (USA). The rabbit anti-MYD88 (ab2064) and rabbit anti-iNOS (ab15323) were purchased from Abcam. Mouse monoclonal anti-TLR4 (76B357.1) and goat anti-Arginase (NB100-59740) were obtained from NOVUS Biologicals. Mouse monoclonal anti-IKBA (L35A5) and mouse monoclonal anti-Beta-actin (8H10D10) were provided by Cell Signaling Technology. Alexa Fluor^®^ 488 donkey anti-mouse, Alexa Fluor^®^ 555 donkey anti-rabbit, and Hoechst 33,342 were obtained from Molecular Probes. LPS-EB ultrapure was acquired from Invivogen. Ham’s F12K medium, puromycin, phosphate-buffered saline (PBS) and fetal bovine serum (FBS) were purchased from Life Technologies (Milan, Italy). Recombinant rat IL-10 was obtained from PeproTech. Nitrocellulose membranes and a Bio-Rad protein assay kit were purchased from Bio-Rad (Marnes La Coquette, France). SuperSignal West Dura chemiluminescent substrate was acquired from Thermo Scientific. Peroxidase-conjugated goat anti-rabbit IgG, peroxidase-conjugated IgG fraction monoclonal mouse anti-goat IgG, light chain specific and peroxidase-conjugated goat anti-mouse IgG were obtained from Jackson ImmunoResearch (West Grove, PA, USA). NucleoSpin^®^ RNA Plus was purchased from Macherey-Nagel. RQ1 RNase-Free DNase was provided by Promega (France), Superscript III Reverse Transcriptase, and PowerUp™ SYBR™ Green Master Mix were obtained from Thermo Fisher Scientific.

### 2.2. Culture of NR8383 PC1/3 KD and NT Cell Lines

PC1/3 knockdown in NR8383 macrophages was performed using lentiviral transduction, as described previously [16]. NR8383 PC1/3 knockdown (PC1/3 KD) and NR8383 non-target (NT) shRNA cell lines were cultured in Ham’s F12K medium supplemented with 15% fetal bovine serum and 12 μg/mL puromycin. The culture was performed at 37 °C in a humidified atmosphere (5% CO_2_). Downregulation of PC1/3 expression has been checked by real time PCR quantification (data not shown).

### 2.3. Immunofluorescence Experiments

Two hundred thousand NR8383 PC1/3 KD and NT cells were grown on a cover slip and treated or not with 200 ng/mL of LPS for 1, 3, and 6 h. The cells were then fixed with 4% paraformaldehyde (PFA) for 10 min. After washes with PBS, cells were permeabilized with 0.2% Triton X-100 for 10 min at room temperature and blocked during 1 h with a blocking solution (PBS 1×, 1% normal donkey serum, 1% BSA, 0.01% Triton). For cell surface staining of TLR4, the permeabilization step was omitted, and cells were directly subjected to the blocking step followed by an overnight incubation at 4 °C with mouse anti-human TLR4 diluted at 10 µg/mL in blocking solution. For intracellular labeling of TLR4 and MYD88, cells were incubated overnight at 4 °C with mouse anti-human TLR4 and rabbit anti-human MYD88 diluted respectively at 20 and 5 µg/mL in blocking solution. After intensive washes with PBS 1×, Alexa Fluor^®^ 555 donkey anti-rabbit and/or Alexa Fluor^®^ 488 donkey anti-mouse diluted at 10 μg/mL in blocking solution was applied for 1 h. After washes with PBS, the nuclei were stained with Hoechst 33,342 (1/10000), and the cells were visualized by confocal microscopy. Fluorescence analysis was conducted using a Zeiss LSM 510 confocal microscope (488 nm excitation for Alexa 488 and 543 nm for Alexa 546) connected to a Zeiss Axiovert 200 M with a 63× 1.4 numerical aperture oil immersion objective. Both channels were excited, collected separately, and then merged to examine the co-localization. The image acquisition characteristics (pinhole aperture, laser intensity, scan speed) were the same throughout the experiments to ensure the comparability of the results.

### 2.4. Total Protein Extraction

Two million NR8383 PC1/3 KD and NT cells were plated on sterile 6-well plates and starved overnight in Ham’s F12K medium supplemented with 2% FBS. For MYD88 detection by Western blot, cells were stimulated with 200 ng/mL of LPS in a serum-free medium during 1, 3, and 6 h or left untreated. For Western blot analysis of IKBA, Arginase and iNOS, cells were treated with 20 ng/mL of IL-10 in serum-free medium during 24 h or left untreated. After stimulation, cells were collected, washed once with ice-cold PBS and then lysed with RIPA buffer (150 mM NaCl, 50 mM Tris, 5 mM EGTA, 2 mM EDTA, 100 mM NaF, 10 mM sodium pyrophosphate, 1% NP40, 1 mM PMSF, and 1× protease inhibitors) for total protein extraction. Sonication of 30 s was performed 3 times and cell debris were removed by centrifugation (20,000× *g*, 10min, 4 °C). Supernatants were collected, and protein concentrations were measured using a Bio-Rad protein assay kit, according to the manufacturer’s instructions.

### 2.5. Western Blot Analysis

Total cell extracts (40 μg) were then analysed by Western blotting. First, proteins were separated by SDS-PAGE electrophoresis and transferred onto nitrocellulose membranes. The membranes were blocked for 1 h at room temperature with PBS-Tween 0.1% + 5% non-fat dry milk (MYD88, iNOS and Arginase) or TBS-Tween 0.1% + 5% non-fat dry milk (IKBA) and incubated overnight at 4 °C with primary antibody diluted in blocking solution. Rabbit anti-MYD88 was diluted at 1 µg/mL, rabbit anti-iNOS at 1/200, goat anti-arginase at 1 µg/mL, and mouse anti-IKBA at 1/1000. The membranes were intensively washed with PBS-Tween 0.1% or TBS-Tween 0.1% and incubated for 1 h at room temperature with peroxidase-conjugated secondary antibody in blocking solution. Goat anti-rabbit was diluted at 0.08 µg/mL, mouse anti-goat at 0.16 µg/mL, and goat anti-mouse at 0.03 µg/mL. Membranes were again washed with PBS-Tween 0.1% or TBS-Tween 0.1%, and proteins were visualized with an enhanced chemiluminescence kit (West Dura from Pierce) according to the manufacturer’s instructions. After this, the membranes were washed with PBS-Tween 0.1% or TBS-Tween 0.1% and stripped for 30 min with 0.2 M citric acid. After intensive washes with TBS- Tween 0.1%, the membranes were blocked for 1 h with TBS-Tween 0.1% + 5% nonfat dry milk. After overnight incubation with mouse anti-Beta-actin diluted at 1/1000 in blocking solution, membranes were washed with TBS- Tween 0.1%. Peroxidase-conjugated goat anti-mouse diluted at 0.005 µg/mL in blocking solution was added for 1 h at room temperature. Membranes were then washed with TBS-Tween 0.1%, and revelation with a chemiluminescence kit was performed. ImageJ software was used to quantify the bands. The results are presented as means ± SD. Normality tests were performed to assess the normal distribution of the data. Data were then analyzed by the Student *t*-test, and values of *p* < 0.05 were considered statistically significant (*p-value of <0.05).

### 2.6. Real Time PCR Quantification

Two million NR8383 PC1/3 KD and NT cells were plated on sterile 6-well plates and starved overnight in Ham’s F12K medium supplemented with 2% FBS. They were then stimulated with 200 ng/mL LPS in serum-free medium or were left untreated. After 0, 1, 3, and 6 h of stimulation, cells were collected, washed once with ice-cold PBS, and RNA was then extracted. RNA of 1 µg was then treated with 1 unit of DNase RQ1 and was retro-transcribed using the SuperScript^®^ III kit. Real time PCR experiments with PowerUp™ SYBR™ Green Master Mix were then performed following the manufacturer’s instructions. The primers used for rat *Tlr4* (Rn.PT.58.11700071) and rat *Myd88* (Rn.PT.58.7022407) were purchased from Integrated DNA Technologies. The *Beta-actin* was used as the reference gene (forward primer: 5′- GCGTCCACCCGCGAGTACAAC -3′; reverse primer: 5′- CGACGACGAGCGCAGCGATA -3′). Real time reactions were conducted on a CFX96 qPCR system (BioRad) using a hot start, then 40 cycles at 94 °C, 3 s; 60 °C, 30 s. Analysis of relative gene expression data was performed using the ΔΔCt method. The results are presented as means ± SD. Normality tests were performed to assess the normal distribution of the data. Data were then analyzed by the Student *t*-test and values of *p* < 0.05 were considered statistically significant (*p-value of <0.05, **p-value of <0.01, ***p-value of <0.001, ****p-value of <0.0001).

### 2.7. Proteomic Analyses

Two million NR8383 PC1/3 KD and NT cells were plated on sterile 6-well plates and starved overnight in Ham’s F12K medium supplemented with 2% FBS. Macrophages were then stimulated or not with 20 ng/mL IL-10 in serum-free medium for 24 h. The next day, macrophages and secretomes were separated by centrifugation. The secretomes were filtered with a 0.22 µm filter to remove cells and analyzed by mass spectrometry. For protein extraction, macrophages were lysed with RIPA buffer (150 mM NaCl, 50 mM Tris, 5 mM EGTA, 2 mM EDTA, 100 mM NaF, 10 mM sodium pyrophosphate, 1% Nonidet P-40, 1 mM PMSF, 1× protease inhibitors). Sonication of 30 s was performed 3 times, and cell debris were removed by centrifugation (20,000× *g*, 10 min, 4 °C). The supernatant containing the proteins were collected for mass spectrometry analyses. A Bradford assay was performed to determine the amount of protein obtained using a Bio-Rad protein assay.

For protein processing, a shotgun bottom-up approach was applied. Thirty µL volume of each sample was prepared after the normalization in the previous step, to obtain a final concentration of 1.5 µg/µL per sample. An equal volume of reduction solution (DTT 0.1 M) was added to each sample then incubated 40 min at 56 °C. Using the FASP method [17], the samples were processed. This technique utilizes a filter with a nominal molecular weight limit of 30,000 (Amicon Ultra-0.5 30k, Millipore). After transferring the samples into the FASP filters, an alkylation step was done using IAA solution (0.05 M) for 20 min in the dark at room temperature. Digestion was then carried overnight at an incubation temperature of 37 °C using LysC/trypsin (40 µg/mL in 50 mM Tris-HCL solution at pH 8). The filters containing the digests were then rinsed using 50 µL of saline solution (0.5 M), and the enzyme activity was stopped with 10 µL of TFA 5% for each tube. Enrichment and desalting were then performed for each sample with a ZipTip C-18 (Millipore) before undergoing LC-MS/MS analysis.

### 2.8. MS Data Acquisition

The analyses were carried out by an Easy-nLC 1000 nano-UPLC chromatographer (Thermo Scientific) interfaced with a Q-Exactive Orbitrap mass spectrometer (Thermo Scientific) with a nano-electrospray ion source. The analysis was performed in reverse phase, and the sample was loaded into a pre-concentration column (75 μm DI × 2 cm, 3 μm, Thermo Scientific). Using an analytical column (Acclaim PepMap C18, 75 μm ID × 50 cm, 2 μm, Thermo Scientific) by applying a linear gradient of acetonitrile in 0.1% formic acid (5% to 35%, for 2 h) at a 300 nL/min flow rate, the peptides were separated. MS analysis was performed by an Orbitrap mass analyzer with a resolution of 70,000 FWHM, a mass range of *m*/*z* 300–1600, an AGC of 3e6 ions, and a maximum injection time of 120 ms. The MS/MS was performed in dependent data mode, defined to analyze the ten most intense ions of MS analysis (Top 10). For MS/MS parameters, the resolution was set to 17,500 FWHM, a mass range of 200–2000 *m*/*z*, an AGC of 5e4 ions, and a maximum injection time of 60 ms. The isolation window was set at 4.0 *m*/*z*.

### 2.9. Data Processing

All data were analyzed by MaxQuant software version 1.5.8.3 [18]. Proteins were identified by comparing all the spectra with the proteome reference database of Rattus norvegicus (Uniprot, release April 2017, 7983 entries, [19]). The digestion parameters were defined using trypsin with 2 maximum missed cleavages. The oxidation of methionine and N-terminal protein acetylation were defined as variable modifications. The carbamidomethylation of cysteine was chosen as fixed modifications. The label-free quantification (LFQ) was done keeping the default parameters of the software. As for initial mass tolerance, 6 ppm was selected for MS mode, and 20 ppm was set for fragmentation data with regard to MS/MS tolerance. The identification parameters of the proteins and peptides were performed with a false discovery rate (FDR) at 1%, and a minimum of 2 peptides per protein in which 1 was unique. The statistical analysis of identified proteins was performed by Perseus software version 1.5.2.6 [20]. The matrix was filtered by removing the potential contaminant, reversed and only identified by site. Then, the samples were grouped into 4 categories: NT, NT-IL-10, KD, and KD-IL-10. Statistical analysis was performed by ANOVA test with a *p*-value of 5%. Hierarchical clustering and profile plot were performed and visualized by Perseus.

### 2.10. Sub-Network Enrichment Pathway Analysis

Using Elseviers’s Pathway Studio (version 11.0//Elsevier), all relationships between the differentially expressed proteins among all conditions were depicted based on the Ariadne ResNet database [21,22]. For proteins identified in NT or KD with or without IL-10 treatment, the subnetwork enrichment analysis (SNEA) algorithm was used to detect the statistically significant altered biological pathways in which the identified proteins are involved. This algorithm uses Fisher’s statistical test to detect any nonrandom associations between two categorical variables organized by a specific relationship. Also, this algorithm starts by creating a central “seed” from all the relevant identities in the database and builds connections with associated entities based on their relationship with the seed. SNEA compares the sub-network distribution to the background distribution using a one-sided Mann–Whitney U-Test, and calculates a *p*-value, thus representing a statistical significance between different distributions. In all analyses that we performed, the GenBank ID was used to form experimental groups based on the different conditions present for analysis. The pathway networks were reconstructed based on biological processes and molecular functions for each single protein, along with its associated targets [23].

## 3. Results

At the cell surface, LPS stimulation of TLR4 leads to the activation of MYD88-dependent signaling and, subsequently, NFκ-B signaling activation [24]. Previously, we found that kinetics of NFKB activation in macrophages was affected by PC1/3 inhibition. PC1/3 KD and NT cells exhibited the same level of NFKB activation after 1 h of LPS stimulation [11]. Conversely, after 3 h of LPS challenge, NFKB activation remained high in PC1/3 KD cells while it decreased in NT cells [11]. These results suggest that the expression of TLR4 at the cell surface may differ between KD and NT macrophages in the time course of an LPS challenge. To test this hypothesis, we performed immunofluorescence experiments to label TLR4 at the cell surface of PC1/3 KD and NT macrophages. 

### 3.1. De Novo Expression of TLR4 at the Cell Surface of Macrophages after Its Internalization is Accelerated in the Absence of PC1/3

**Figure 1:**
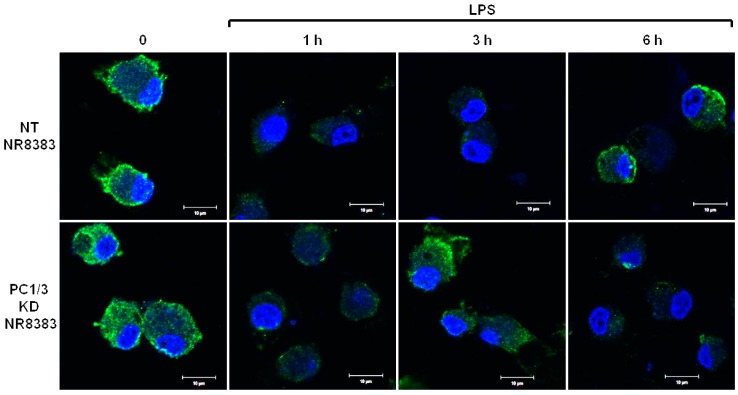
After its internalization, TLR4 is more quickly re-expressed at the plasma membrane of PC1/3 KD macrophages challenged with LPS. NT and PC1/3 KD NR8383 macrophages were treated with 200 ng/mL of LPS for 0, 1, 3, and 6 h. Cells were then fixed and stained with an antibody directed against the extracellular domain of TLR4 (green). To label TLR4 at the cell surface, no permeabilization step was performed. The nuclei were counterstained with Hoechst 33,342 (blue). Confocal microscopy analysis was then performed. Bar = 10 μm (*n* = 3).

PC1/3 KD and NT macrophages were stimulated with 200 ng/mL of LPS during 1, 3, and 6 h. To detect TLR4 at the cell surface, no permeabilization step was performed and an antibody directed against the extracellular domain of the receptor was used. Three independent experiments were performed and revealed that kinetics of TLR4 expression at the cell surface was altered in PC1/3 KD cells. Such an alteration is presented in Figure 1. In NT macrophages, expression of TLR4 at the cell surface decreased strongly after 1 h of LPS stimulation. This indicates its internalization in early endosomes. TLR4 level at the plasma membrane remained very low at 3 h after LPS challenge and the receptor was again expressed at the cell surface after 6 h of stimulation. In KD cells, TLR4 was also internalized after 1 h of LPS treatment. However, at 3 h post LPS treatment, the receptor was again clearly observed at the plasma membrane. After 6 h of LPS treatment, TLR4 again disappeared from the cell surface. These results demonstrate that in the absence of PC1/3, the turnover of TLR4 expression at the plasma membrane is altered. 

### 3.2. PC1/3 is Involved in the Control of TLR4 Trafficking

**Figure 2:**
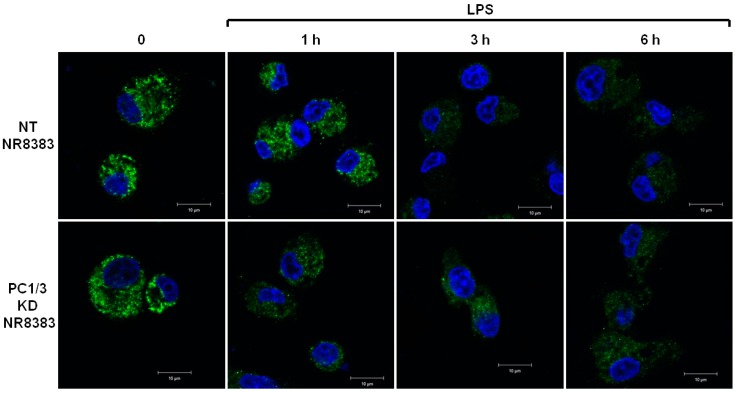
PC1/3 involvement in the control of TLR4 trafficking. NT and PC1/3 KD NR8383 macrophages were treated with 200 ng/mL of LPS for 0, 1, 3, and 6 h. Cells were then fixed, permeabilized and stained with an antibody directed against the extracellular domain of TLR4 (green). The nuclei were counterstained with Hoechst 33,342 (blue). Confocal microscopy analysis was then performed. Bar = 10 μm.

The differences of TLR4 expression at the cell surface observed in the absence of PC1/3 may reflect an alteration of TLR4 intracellular trafficking. To test this hypothesis, immunofluorescence experiments were conducted to follow the intracellular trafficking of TLR4 in PC1/3 KD and NT macrophages. Three independent experiments were performed and revealed that intracellular trafficking of TLR4 was altered in PC1/3 KD cells. Such an alteration is presented in Figure 2. In resting NT cells, intracellular TLR4 was detected as marked aggregates. The aggregates were still visible after 1 h of LPS challenge. On the contrary, after 3 and 6 h of LPS treatment, the intensity of TLR4 staining decreased strongly. This may reflect the trafficking of the receptor towards the cell surface for its re-expression at 6 h post LPS treatment, as observed in Figure 1. In KD cells, intracellular TLR4 was also observed as marked aggregates in resting macrophages (Figure 2). 

However, the intensity of the aggregates diminished strongly after 1 h of LPS stimulation and remained weak in the remaining time course of the experiment. This is also in correlation with the quicker re-expression of the receptor at the plasma membrane in KD cells (Figure 1). These results demonstrate that PC1/3 is involved in the control of TLR4 trafficking from intracellular compartments towards the plasma membrane. 

### 3.3. The Levels of Tlr4 mRNA Decrease in NT and PC1/3 KD Macrophages Challenged with LPS

**Figure 3:**
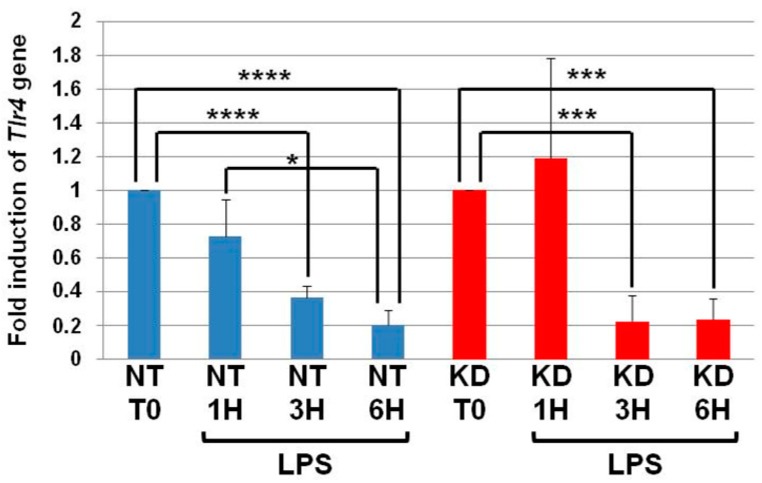
The levels of *Tlr4* mRNA decrease in NT and PC1/3 KD macrophages challenged with LPS. NT and PC1/3 KD cells were challenged with 200 ng/mL of LPS for 0, 1, 3, and 6 h. The results are obtained from three independent experiments and depicted through graphic representations (means ± SD). Data were analyzed by the Student *t*-test and values of *p* < 0.05 are considered statistically significant (*p-value of <0.05, ***p-value of <0.001, ****p-value of <0.0001).

NT and PC1/3 KD cells were exposed to LPS for 1, 3, or 6 h, and quantitative RT-PCR experiments were performed (Figure 3). In NT cells, the level of *Tlr4* messengers decreased in the time course of LPS treatment and was significantly lower at the third and sixth hour of the challenge. Similar results were observed in the PC1/3 KD macrophages. This suggests that the receptor can be synthesized from a pre-existing pool of messengers. This may support the de novo expression of the receptor at the cell surface or the replenishment of TLR4 stock in the endosomal recycling compartment (ERC) if the receptor translocated from this compartment. Conversely, this decrease may also reflect the degradation of the messengers to block the production of the receptor. In any case, since the level of *Tlr4* messengers displays the same modulation in NT and PC1/3 KD cells, we can conclude that the difference observed between the two types of cells in Figure 1 and Figure 2 only relies on the alteration of TLR4 trafficking.

### 3.4. MYD88, the Key TLRs Signalling Adaptor, Displays a Potential Cleavage Site for Proprotein Convertases in Its TIR Domain

**Figure 4:**
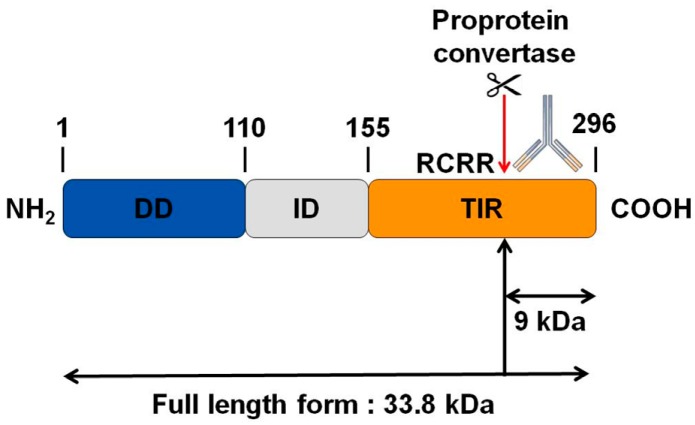
Bioinformatics analysis revealing a potential cleavage site for proprotein convertases in the C-terminal part of MYD88. While the molecular weight of MYD88’s full-length form is 33.8 KDa, such cleavage could release a C-terminal fragment of 9 KDa. The antibody used in the corresponding Western blot experiments is directed against the C-terminal part of MYD88. Thus it can detect MYD88’s full-length form and the cleaved C-terminal fragment of 9 KDa. DD: death domain; ID: intermediate domain.

Interestingly, by bioinformatics analysis, we found a potential cleavage site for proprotein convertases in the TIR domain of MYD88 (Figure 4). We thus hypothesize that PC1/3 may downregulate this pathway through the cleavage of MYD88. 

### 3.5. Study of MYD88 Localization in NT and PC1/3 KD Macrophages Challenged with LPS

**Figure 5:**
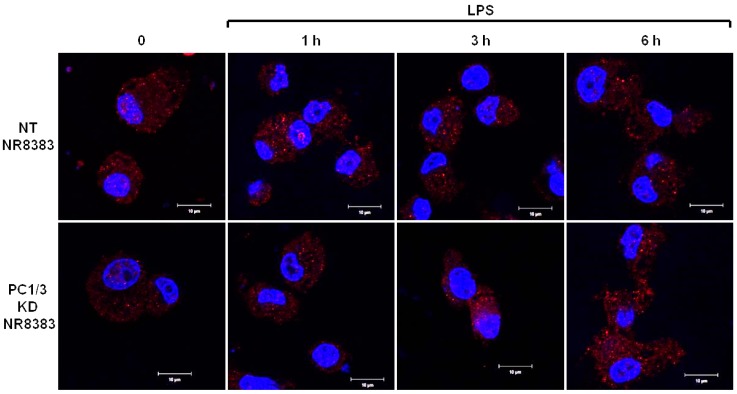
PC1/3 does not control MYD88 localization. NT and PC1/3 KD NR8383 macrophages were treated with 200 ng/mL of LPS for 0, 1, 3, and 6 h. Cells were then fixed, permeabilized, and stained with an antibody directed against the C-terminal part of MYD88 (red). The nuclei were counterstained with Hoechst 33,342 (blue). Confocal microscopy analysis was then performed. Bar = 10 μm.

As a first step, we studied if the inhibition of PC1/3 in macrophages impacted MYD88 localization. To achieve this goal, NT and PC1/3 KD NR8383 cells were stimulated with 200 ng/mL of LPS during 1, 3, and 6 h. Immunofluorescence experiments were then conducted to stain MYD88 (Figure 5). 

During the time course experiments, MYD88 was detected as aggregates distributed inside the cytoplasm of NT and PC1/3 KD macrophages. No differences between NT and KD cells were observed, indicating that MYD88 localization was not affected by the absence of PC1/3.

### 3.6. PC1/3 KD Cells Exhibit Higher Levels of MYD88 than NT Cells after 6 h of LPS Challenge

**Figure 6:**
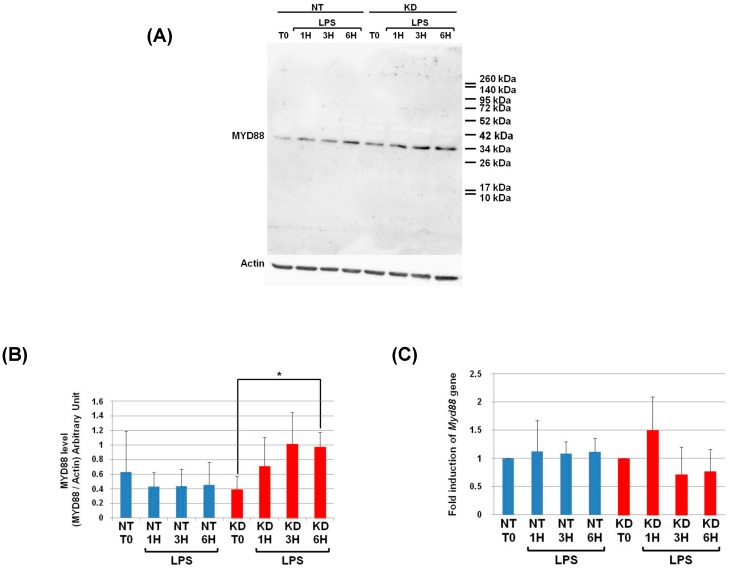
At 6 h post-LPS treatment, PC1/3 KD cells significantly exhibit higher levels of MYD88 than NT cells. (**A**) NT and PC1/3 KD NR8383 macrophages were treated with 200 ng/mL of LPS for 0, 1, 3, and 6 h. Western blot studies were then conducted with anti-MYD88, see Figure 4. (**B**) Intensities of MYD88’s full-length form at 33.8 KDa, quantified and normalized to those of Actin. (**C**) The modulation of *Myd88*gene expression investigated in NT and PC1/3 KD NR8383 macrophages challenged with 200 ng/mL of LPS for 0, 1, 3, and 6 h. The results are obtained from three independent experiments and depicted through graphic representations (means ± SD). Data are analyzed by the Student *t*-test, and values of *p* < 0.05 are considered statistically significant (*p-value of <0.05).

As a next step, we examined if PC1/3 controlled MYD88 cleavage. To test this hypothesis, NT and PC1/3 KD NR8383 cells were challenged with 200 ng/mL of LPS during 1, 3, and 6 h. Then proteins were extracted and Western blotting was performed with an antibody directed against the C-terminal part of MYD88 (Figure 4 and Figure 6A). If MYD88 is cleaved at the potential cleavage site for proprotein convertases, a fragment of 9KDa will be released (Figure 4). However, after the time-course experiments, this fragment was not observed in NT or KD cells (Figure 6A). Only the full-length form of MYD88 at 33.8 KDa was observed. The intensity of this band was quantified, and this revealed that changes in MYD88 levels during the LPS challenge displayed a different trend between NT and PC1/3 KD cells (Figure 6B). In NT macrophages, the amount of MYD88 tended to diminish slightly in the time-course of the experiments. However, this decrease was not significant. On the contrary, in PC1/3 KD cells treated with LPS, the level of MYD88 displayed a tendency to increase from 1 to 3 h after the challenge, and this trend became more obvious at the sixth hour after treatment. Altogether, these results show that PC1/3 does not control MYD88 cleavage but may impact its biosynthesis. 

### 3.7. Measurement of Myd88 mRNA Levels in NT and PC1/3 KD Macrophages Challenged with LPS

To enable this increase of MYD88 levels after LPS treatment (Figure 6B), we hypothesized that *Myd88* gene expression could be induced in KD cells. To test this hypothesis, NT and PC1/3 KD cells were exposed to LPS for 1, 3, or 6 h, and quantitative RT-PCR experiments were performed (Figure 6C). Surprisingly, neither KD cells nor NT cells exhibited a significant modulation of *Myd88* gene expression. Moreover, the levels of *Myd88* messengers were not different between NT macrophages and KD macrophages. Therefore, a clear correlation between protein synthesis and gene expression in KD cells treated with LPS cannot be made. This suggests that another mechanism such as augmentation of protein half-life or stability could be responsible for this increase. 

### 3.8. PC1/3 KD Macrophages Exhibit Resistance to Pro-Tumoral Effect of IL-10 Treatment

Previously, we found that the anti-inflammatory STAT3 pathway was repressed in PC1/3 KD macrophages [11,12,13]. It is noteworthy that this effect persisted even in the presence of Interleukin-10 treatment [11,13]. This suggests that PC1/3 KD cells may be resistant to an immunosuppressive environment. In order to test this hypothesis, NT and PC1/3 KD cells were incubated for 24 h with IL10 and a proteomics study was performed. The extracted peptides were subjected to shotgun analysis followed by label-free quantification. There were 1439 proteins identified. However, only 309 proteins showed a significant difference of expression based on LFQ values after subjecting the data to multiple ANOVA tests with *p*-value = 0.05. In this context, 240 specific proteins were identified, i.e., 51 in NT control, 139 in NT cells treated with IL10, 20 in KD control, and 30 in KD cells treated with Il-10. Among the 20 specific proteins identified in PC1/3 KD macrophages control, pirin a transcriptional co-regulator of NFKB was found (Appendix A). As represented in the string analyses associated with sub-network enrichment (Figure S1A), microphthalmia-associated transcription factor (MITF), the hepatocyte growth factor receptor as well as transcription factors (survival of motor neuron-related-splicing factor 3, cleavage stimulation factor subunit 1, protein RTF2 homolog; replication termination factor 2 domain containing 1) were also characterized in PC1/3 KD control cells. In PC1/3 KD cells treated with IL-10 (Figure S1B), some of the proteins identified were related to the MAPK signaling pathway (ras GTPase-activating protein 1, mitochondrial fission regulator 1-like, serine/threonine-protein kinase A-Raf, regulator complex protein LAMTOR3) and organelle organisation (ribosome biogenesis protein BOP1, nuclear pore complex protein Nup153, mitochondrial rho GTPase 2, ezrin, vacuolar protein sorting-associated protein 33A, zinc finger protein 83, coiled-coil domain-containing protein 39, mitochondrial fission regulator 1-like, ras-related protein Rab-7L1). In NT cells treated with IL-10, the string protein interaction analysis (Figure S1C) revealed that the proteins were in KEGGS pathways related to prostate, endometrial, and colorectal cancers as well as acute myeloma leukemia. Reactome analyses (data not shown) showed that the contact of these NT cells treated with IL-10 was implicated in the Gastrin-CREB signaling pathway, NCAM signaling, negative regulation of MAPK pathways, or NTRK1 signaling. These results established that NT and PC1/3 KD cells did not respond in the same way after IL-10 treatment. Compare to NT cells, KD macrophages were more resistant to the modifications induced by IL-10. These results were reinforced by proteomic performed on the secreted factors from these cells, which confirmed the precedent results (Appendix A). In fact, NT cells treated with IL-10 produced tumor activator factors such as RhoA, the glia maturation factor beta, galectin-1, the isocitrate dehydrogenase subunit alpha, and the D-3-phosphoglycerate dehydrogenase (Figure S2). By contrast, PC1/3 KD cells treated with IL-10 did not produce such tumor growth factors (Appendix A). Reactome analyses revealed proteins involved in biosynthesis and metabolism of amino acids and their derivatives, iron uptake and transport as well as the 2-oxocarboxylic acid metabolism. Compared to NT cells (Figure S3A,B), subnetwork analyses confirmed that PC1/3 KD cells were engaged in inflammatory response even in the presence of IL-10 (Figures S3C,D). In the presence of IL-10, NT cells switched to cells invasion, spreading and migration (Figure S3B), whereas KD cells were still in inflammatory pattern with inhibition of apoptosis, cells differentiation, and proliferation (Figure S3D).

**Figure 7:**
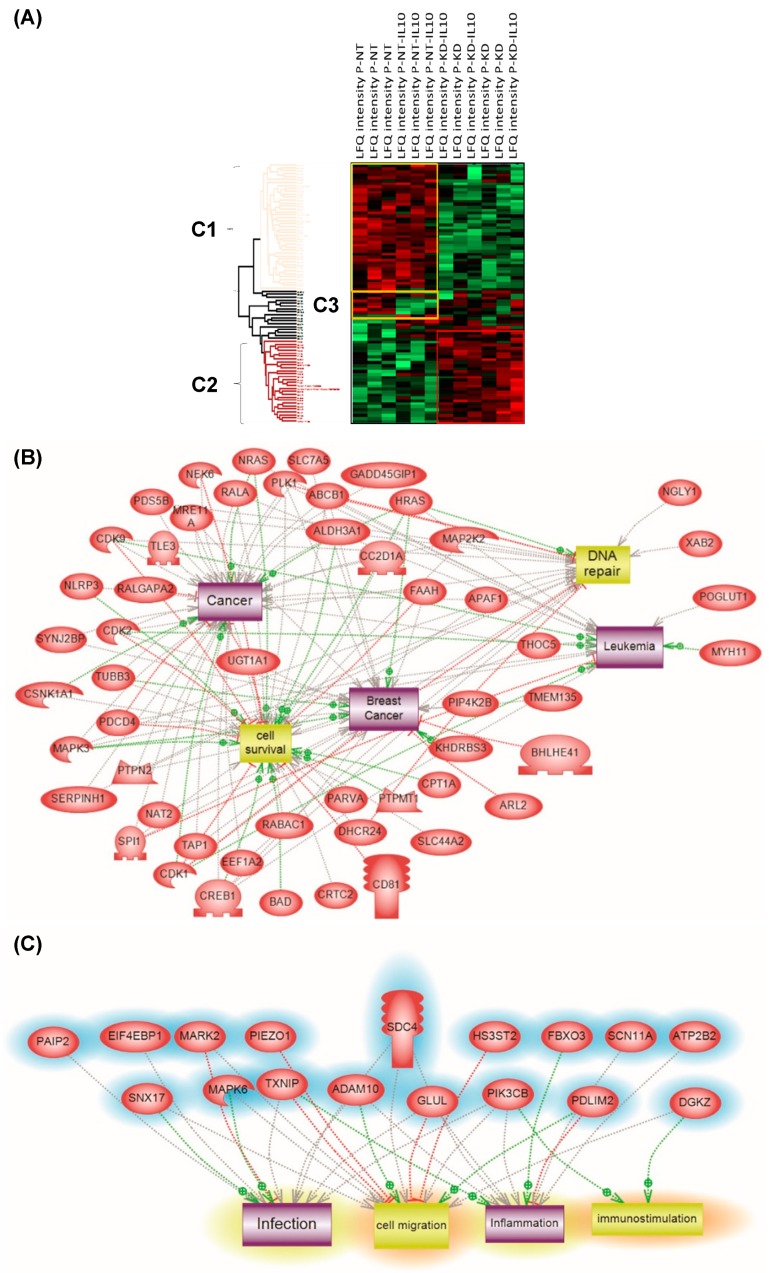
(**A**) Analysis of the global proteome of PC1/3KD macrophages stimulated with IL-10. NR8383 control (NT) and PC1/3 (KD) macrophages were stimulated (NT-IL10 or KD-IL10) or not with 20 ng/mL of IL-10 for 24 h. The cells were lysed and digested by trypsin during FASP (amicon 30 kDa). The peptides were analysed by mass spectrometry. Protein identification was done using MaxQuant software and then analysed on Perseus. A Heatmap was generated to visualize the differences in relative protein quantities between macrophages under the different conditions. 4 clusters were supervised. Bands C1–C3 corresponds to subnetwork analyses. (**B**) corresponds to Cluster 1 and (**C**) to Cluster 2. The results were obtained from three independent experiments.

Principal component analysis (PCA) was applied to the 309 quantified proteins in NT and PC1/3 KD cells treated or not with IL-10. Three different clusters were identified and two blocks were observed (Cluster 1 and Cluster 2) (Figure 7A). Cluster 1 corresponded to NT cells and Cluster 2 to PC1/3 KD cells with or without IL-10 treatment. NT control cells and NT cells treated with IL-10 were well regrouped. In the majority, the proteins identified were the same, except for a few proteins in Cluster 3 which were overexpressed in NT control macrophages and downregulated in NT macrophages stimulated with IL-10. Cluster 1 revealed proteins involved in metabolism, especially in pyruvate metabolism or glycolysis and in apoptosis (Figure S4). In contrast, the proteins identified in PC1/3 KD cells, treated or not with IL-10 (Cluster 2), were mostly involved in cellular processes (Figure S5). 

Furthermore, some specific proteins involved in immune response or some anti-oncogenic factors were observed, e.g,. CD166, CD9, CD36, and the Malignant T-cell-amplified sequence 1. CD166 is known to promote T-cell activation and proliferation via its interactions with CD6. The tetraspanin CD9 is expressed by all leukocytes, especially macrophages, and plays pivotal roles in their physiology. The scavenger receptor CD36, is known to interact with TLR4-TLR6 in sterile inflammation as we previously demonstrated in PC1/3 KD cells using a paclitaxel drug [13]. Again, PC1/3 KD cells even in the presence of IL-10 clearly showed a pro-inflammatory response compared to NT cells, which expressed a pro-tumoral profile in the presence of IL-10. Subnetwork analyses confirmed that proteins involved in Cluster 1 expressed a cancerous phenotype (Figure 7B) whereas the ones in Cluster 2 were more representative of an inflammatory phenotype (Figure 7C).

**Figure 8:**
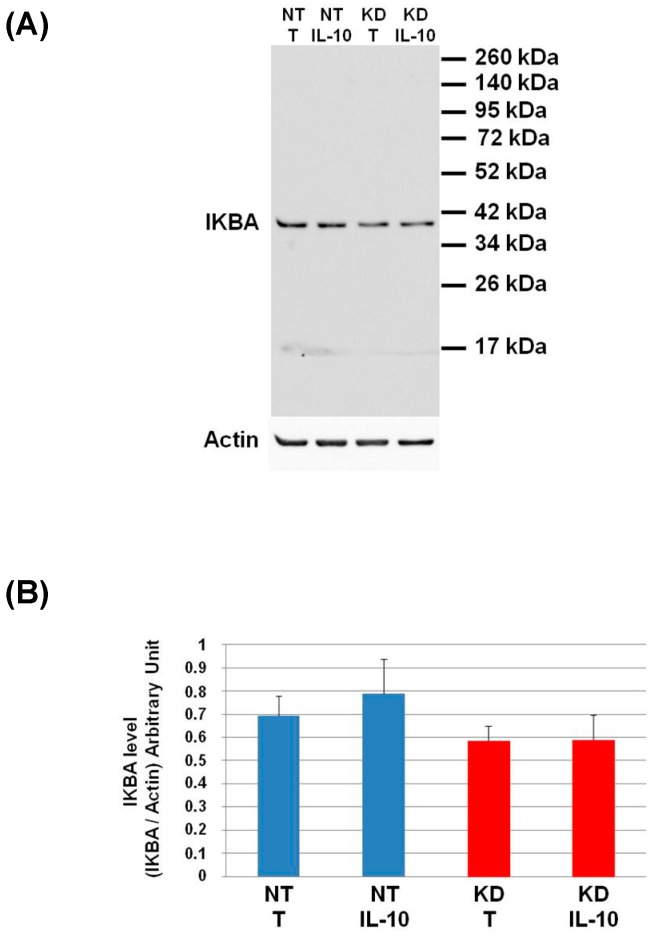
Study of IKBA degradation after IL-10 treatment. (**A**) NT and PC1/3 KD NR8383 macrophages were treated with 20 ng/mL of IL-10 for 24 h. Western blot studies were then conducted with anti-IKBA. (**B**) Intensities of IKBA quantified and normalized to those of Actin. The results are obtained from three independent experiments and depicted through graphic representations (means ± SD). Data are analyzed by the Student *t*-test. No significant differences were found.

To validate the pro-inflammatory phenotype of PC1/3 KD macrophages even under IL-10 treatment, we studied the activation of the pro-inflammatory pathway NFKB. For this purpose, we analyzed by Western blot the degradation of IKBA (Figure 8).

**Figure 9:**
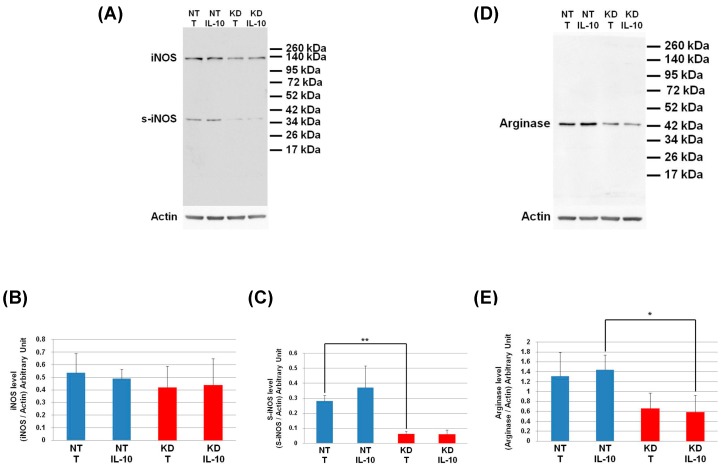
Under IL-10 treatment, PC1/3 KD cells exhibit lower levels of arginase than NT cells. NT and PC1/3 KD NR8383 macrophages were treated with 20 ng/mL of IL-10 for 24 h. (**A**) Western blot studies conducted with anti-iNOS. (**B**) Intensities of iNOS quantified and normalized to those of Actin. (**C**) Intensities of s-iNOS quantified and normalized to those of Actin. (**D**) Western blot studies conducted with anti-arginase. (**E**) Intensities of arginase quantified and normalized to those of Actin. The results are obtained from three independent experiments and depicted through graphic representations (means ± SD). Data are analyzed by the Student *t*-test and values of *p* < 0.05 are considered statistically significant (*p-value of <0.05, **p-value of <0.01).

The level of IKBA had the tendency to be lower in KD cells than in NT cells and even more after IL-10 treatment. This shows that the IL-10 cytokine does not affect the NFKB signalling pathway in KD cells. We then decided to assess by Western blot, the levels of Arginase and iNOS in NT and PC1/3 KD macrophages challenged or not with IL-10 for 24 h. These two enzymes regulate the production of nitric oxide (NO), another factor indicative of an inflammatory reaction These experiments revealed that the iNOS levels were equal between KD and NT cells treated or not with IL-10 (Figure 9A,B). However, surprisingly, the Western blot also revealed the existence of a truncated form of iNOS named s-iNOS, which displayed a MW closed to 38 KDa (Figure 9A). Interestingly, we found that the levels of s-iNOS showed a clear trend to be lower in KD cells compared to NT cells whatever the conditions (Figure 9C). The same tendencies were observed for arginase levels (Figure 9D,E). Altogether, these results reinforce the hypothesis that KD cells are resistant to IL-10 inhibitory treatment. Thus, KD cells seem to escape the inhibitory effect of IL-10 and the pro-inflammatory pathway is not affected. 

## 4. Discussion

**Figure 10:**
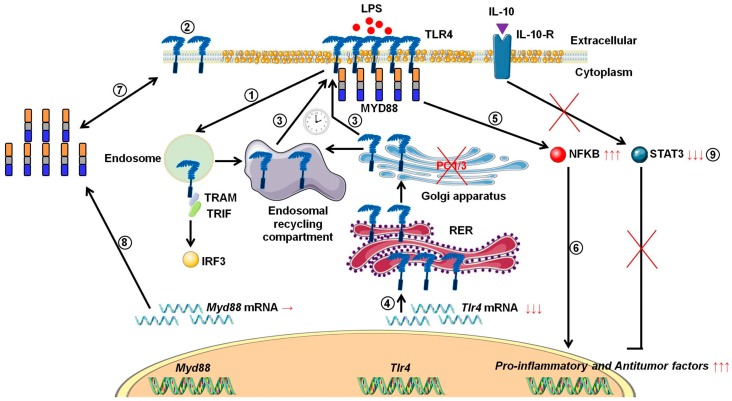
Proposed model depicting the impact of PC1/3 knockdown on the activation of TLR4. Yellow droplets in the cell membrane symbolize that KD cells exhibited modifications of their lipid profile. (**1**) After LPS treatment, TLR4 is internalized in the endosomes to trigger the MYD88-independent signaling resulting in the activation of IRF-3. (**2**) As a result, the level of TLR4 at the plasma membrane decreases. (**3**) In the absence of PC1/3, TLR4 is more quickly re-expressed at the cell surface as symbolized by the clock pictogram, i.e,. 3 h after LPS treatment. These receptors may translocate from endosomal recycling compartment (ERC) where they are stored or from the Golgi apparatus after their synthesis and maturation. (**4**) TLR4 receptor can be synthesized from a pre-existing pool of messengers. This may support the de novo expression of the receptor at the cell surface or the replenishment of TLR4 stock in ERC if the receptor translocated from this compartment. (**5**) The re-expression of TLR4 at the cell surface after 3 h of LPS challenge in PC1/3 KD macrophages leading to a stronger activation of the pro-inflammatory NFKB pathway while the anti-inflammatory STAT3 pathway is downregulated. (**6**) As a consequence, PC1/3 KD macrophages stimulated with LPS secrete more pro-inflammatory cytokines and antitumor factors. After treatment of PC1/3 KD cells with LPS during 6 h, TLR4 is again internalized, and its amount at the cell surface decreased (2). (**7**) The increase of MYD88 level observed in PC1/3 KD macrophages after 6 h of LPS treatment may compensate the decrease of TLR4 at the cell surface and maintain the activation of the signaling pathway. (**8**) A clear correlation between protein synthesis and gene expression in KD cells treated with LPS can not be made. Therefore, another mechanism, such as augmentation of protein half-life or stability, may be responsible for this increase. (**9**) PC1/3 KD cells resist to the inhibitory effect of IL-10 and clearly show a pro-inflammatory response.

During tumor development, macrophages are recruited and orientated towards the pro-tumoral phenotype. As a result, these macrophages exert pro-tumoral functions [8]. At present one of the main anti-tumoral strategies consists in re-polarizing the TAMs towards a pro-inflammatory phenotype [9,10] in order to boost the immune response in cancer patients. In this context, we previously demonstrated that combining the inhibition of PC1/3 and TLR4 activation in macrophages could be a very promising anti-tumoral immunotherapy [11,13,14]. Indeed, PC1/3 knock down macrophages challenged with TLR4 ligands such as LPS or paclitaxel display pro-inflammatory and anti-tumoral phenotypes [11,13,14]. At the cell surface, LPS stimulation of TLR4 leads to the activation of MYD88-dependent signaling and, subsequently, NFKB activation [24]. TLR4 is then internalized in early endosomes [25] and triggers the MYD88-independent signaling resulting in the activation of interferon response factor (IRF) 3 [26]. One of the main features of PC1/3 KD macrophages is a stronger activation of the pro-inflammatory pathway NFKB after 3 h of TLR4 stimulation with LPS [11]. This suggests that desensitization of TLR4 signalling pathway is reduced in PC1/3 KD macrophages. It was demonstrated that ectodomain shedding of TLR4 in human aortic endothelial cells downregulated the activity of the receptor [27]. TLR4 ectodomain is cleaved by disintegrin and metalloprotease 17 (ADAM17), a known substrate of PC1/3 [28]. Therefore, cleavage of the TLR4 extracellular domain would decrease in PC1/3 KD cells. However, at 1 h after the LPS challenge, the detection of TLR4 at the cell surface decreased strongly in both NT and KD cells (Figure 1). Therefore, our present study enables the exclusion of this hypothesis. It also showed that there was no default in TLR4 internalization in KD cells. This is consistent with our previous data demonstrating that IRF-3 activation was identical in PC1/3 KD and NT macrophages challenged with LPS for 1, 3, and 6 h [11]. Conversely, our results revealed that the stronger NFKB activation observed in KD cells stimulated during 3 h with LPS relied on the quicker re-expression of the receptor at the cell surface (Figure 1). This was correlated with an alteration of TLR4 intracellular trafficking (Figure 2), as we already described for TLR9 [12]. During LPS treatment of NR8383 macrophages, PC1/3 trafficked with TLR4 [16]. Since PC1/3 is mainly located in the trans-Golgi network [16], our results suggest that TLR4 translocation from the Golgi apparatus to the plasma membrane can be restrained. Such an inhibition can therefore be removed in PC1/3 KD macrophages (Figure 1 and Figure 2). However, we cannot also exclude that the receptors re-expressed at the cell surface may originate from ERC where they translocated after their internalization or their maturation in the Golgi apparatus [29,30]. This is consistent with the detection of intracellular TLR4 as aggregates in resting NT and KD macrophages and their scattering in time course of LPS treatment (Figure 2). While this was observed after 3 and 6 h of LPS challenge in NT cells, it occurred as soon as the first hour of treatment in KD cells. This suggests that the recycling process can be sustained in KD macrophages and can explain why TLR4 was again detected at the cell surface after 1 h of LPS stimulation (Figure 1). Our previous findings also support this hypothesis [11]. We found that the endosomal compartment was remodeled in KD cells. In accordance with this observation, the expression of several specific trafficking regulators RAB GTPases, such as RAB11, was increased. Indeed, RAB11 controls the localization of TLR4 in the ERC [29] and the recycling of proteins at the cell surface [31,32]. Apart from these hypotheses, another possibility can also be considered. It has been demonstrated that the cytoskeleton can modulate the intracellular trafficking machinery through microtubules and calcium mobilization [33,34]. Previously, we reported profound perturbations of cytoskeletal organization and calcium homeostasis in KD cells [11]. Therefore, these alterations may be responsible or contribute to the modifications of TLR4 trafficking in KD cells. To control TLR4 expression at the cell surface, PC1/3 may cleave specific substrates. It has been shown that ATP-binding cassette sub-family A member 1 (ABCA1) is involved in the inhibition of TLR4 expression at the cell surface [35]. ABAC1 promotes cholesterol efflux from macrophages [35,36]. It also exhibits several potential cleavage sites for proprotein convertases in its sequence, as revealed by bioinformatics analysis (data not shown). Interestingly, using water-assisted laser desorption/ionization mass spectrometry technology [37], we found that KD cells also exhibited modifications of its lipid profile (unpublished results). It is therefore tempting to speculate that PC1/3 may also regulate TLR4 trafficking through the processing of ABCA1. In any case, only the identification of PC1/3 substrates will clarify how this endopeptidase regulates TLR4 trafficking and/or recycling. During the present study, we also observed that TLR4 was again internalized in KD cells treated during 6 h with LPS while it was re-expressed at the cell surface of NT cells (Figure 1). A stronger activation of NFKB pathway could have been expected in NT cells. However, at this time point, NFKB activation was the same in NT and KD macrophages [11]. The increase of MYD88 level observed in PC1/3 KD macrophages (Figure 6B,C) may compensate the decrease of TLR4 at the cell surface and maintain the activation of the signaling pathway. In Figure 10, we propose a model to recapitulate the main changes displayed by PC1/3 KD macrophages.

## 5. Conclusions

Altogether, these results show that the length of TLR4 desensitization is reduced in PC1/3 KD macrophages. Even in the presence of the inhibitor cytokine IL-10, PC1/3 KD cells have clearly also shown their resistance to the pro-tumoral orientation. The NFKB pathway is not modulated by the IL-10 STAT3 pathway and KD cells still produce innate immune pro-inflammatory factors such as the CCL3, galectin 3, caspase 8, the bone marrow stromal antigen 2, tax1-binding protein 1 homolog, oncomodulin, HMGB1, the anti-oncogene malignant T-cell-amplified sequence and express several specific pro-inflammatory proteins (CD48, C166, CD44). In the context of antitumoral immunotherapy, these characteristics are of utmost importance since TLR4 can be repeatedly activated in macrophages inhibited for PC1/3, even in an anti-inflammatory environment. This frequent activation of the receptor may reverse the phenotype of TAMs and enable their reactivation to boost the immune system in cancer patients.

## Supplementary Materials

The following are available online at https://www.mdpi.com/2073-4409/8/12/1490/s1, **Figure S1**: String analyses associated with sub-network enrichment deduced from shotgun proteomic analyses of NT and PC1/3 KD NR8383 macrophages treated or not with 20 ng/mL of IL-10 for 24 h, **Figure S2**: String analyses associated with sub-network enrichment deduced from shotgun proteomic analyses of secretome from NT NR8383 macrophages treated with 20 ng/mL of IL-10 for 24 h, **Figure S3**: Sub-network analyses of specific proteins identified in (**A**) NT control cells, (**B**) NT cells treated with IL10, (**C**) PC1/3 KD control cells, (**D**) PC1/3 KD cells treated with IL10, **Figure S4**: The string protein analysis revealed interactions of the proteins identified in Cluster 1 after shot gun proteomic studies of NT cells treated with IL-10, **Figure S5**: The string protein analysis revealed interactions of the proteins identified in Cluster 2 after shot gun proteomic studies of PC1/3 KD cells treated with IL-10.

## Appendix A

List of the specific proteins identified in cell extracts and secretomes of NT and PC1/3 KD NR8383 macrophages treated or not with 20 ng/mL of IL-10 for 24 h.
