# Supplementary figures and images for: PC1/3 KD Macrophages Exhibit Resistance to the Inhibitory Effect of IL-10 and a Higher TLR4 Activation Rate, Leading to an Anti-Tumoral Phenotype
Source: Cells. 2019 Nov 22;8(12):1490. doi: 10.3390/cells8121490 (PMC6953035; doi:10.3390/cells8121490)

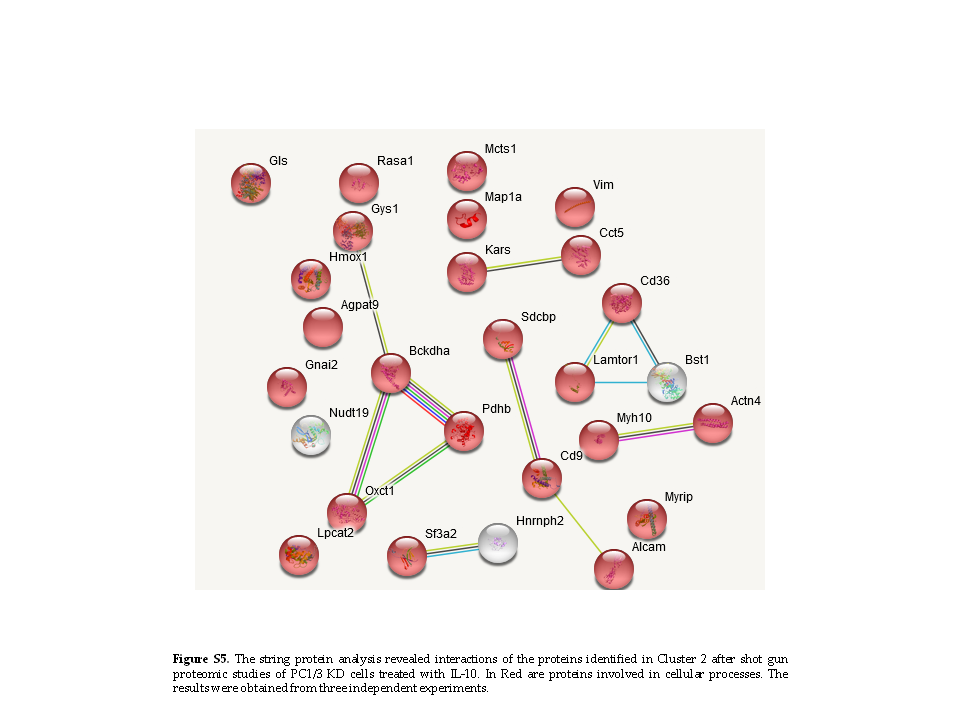

Supplement: Supplementary file 1 [file cells-08-01490-s001.zip › Figure S5 Rodet et al., 2019.tif]

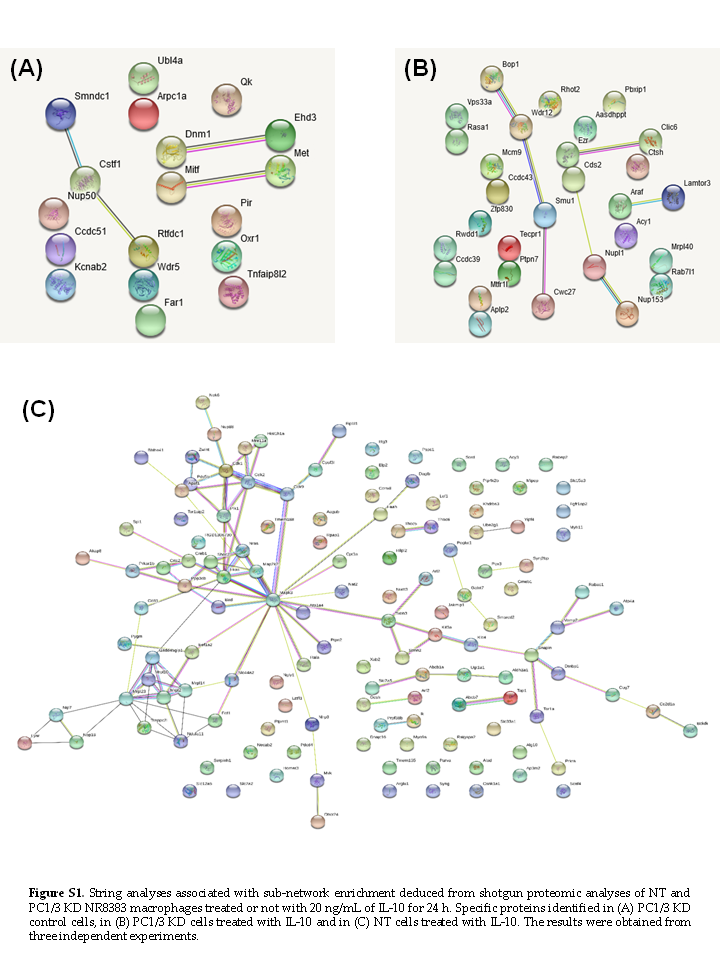

Supplement: Supplementary file 1 [file cells-08-01490-s001.zip › Figure S1 Rodet et al., 2019.tif]

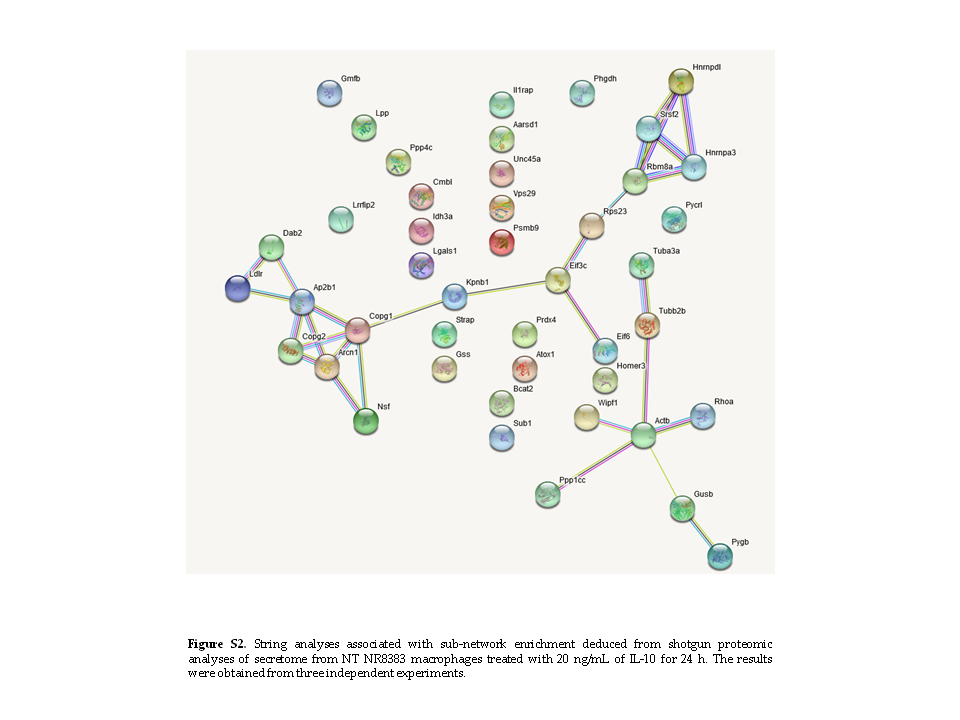

Supplement: Supplementary file 1 [file cells-08-01490-s001.zip › Figure S2 Rodet et al., 2019.tif]

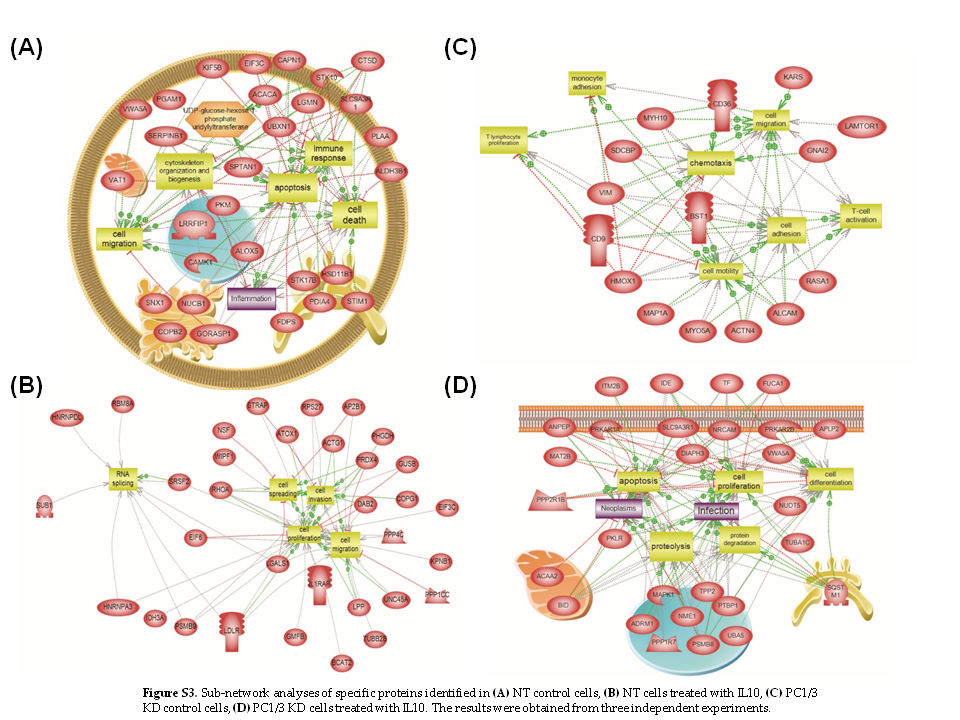

Supplement: Supplementary file 1 [file cells-08-01490-s001.zip › Figure S3 Rodet et al., 2019.tif]

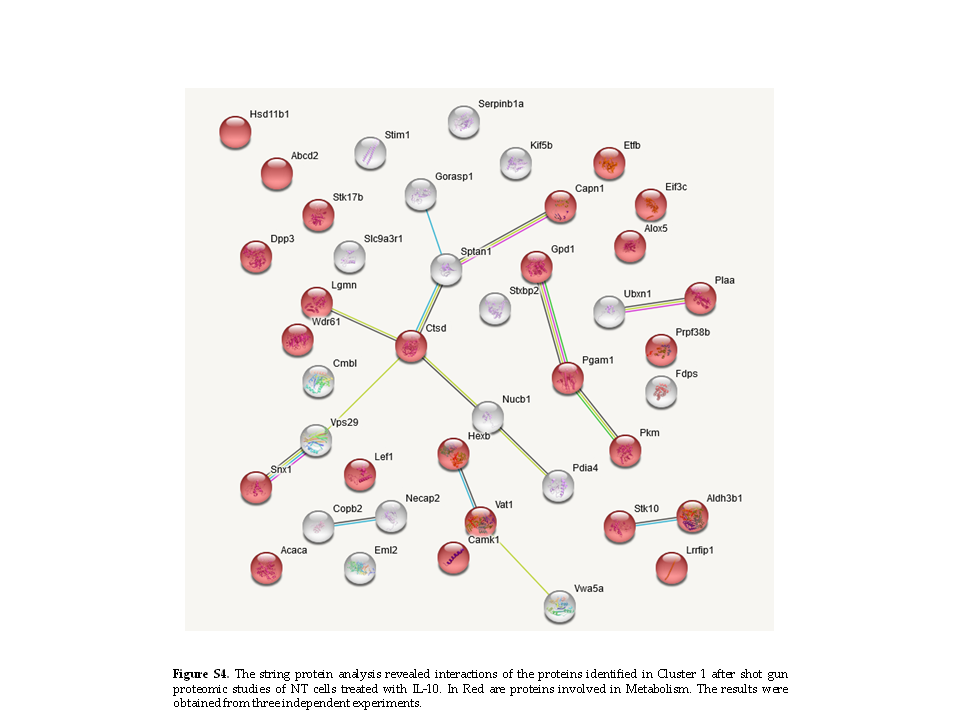

Supplement: Supplementary file 1 [file cells-08-01490-s001.zip › Figure S4 Rodet et al., 2019.tif]
